# Simultaneous liver kidney transplantation and (bilateral) nephrectomy through a midline is feasible and safe in polycystic disease

**DOI:** 10.1371/journal.pone.0174123

**Published:** 2017-03-17

**Authors:** Ina Jochmans, Diethard Monbaliu, Laurens J. Ceulemans, Jacques Pirenne, Jiri Fronek

**Affiliations:** 1 Abdominal Transplant Surgery, University Hospitals Leuven, Leuven, Belgium; 2 Department of Microbiology and Immunology, Lab of Abdominal Transplant Surgery, KU Leuven, Leuven, Belgium; 3 Transplant Surgery Department, Institute for Clinical and Experimental Medicine, Prague, Czech Republic; Istituto Mediterraneo per i Trapianti e Terapie ad Alta Specializzazione, ITALY

## Abstract

In Eurotransplant, 50% of simultaneous liver kidney transplantations (SLK) are performed for polycystic disease. Classically, liver and kidney are transplanted in two steps: liver through a subcostal incision, kidney through a separate oblique incision. Liver and kidney volume can make this ‘two-step’ procedure challenging, especially if simultaneous native nephrectomy is indicated. A ‘one-step’ SLK through a xiphopubic laparotomy might be a safe alternative, facilitating mobilization of the voluminous polycystic liver and native nephrectomy whilst offering access to iliac fossae for kidney transplantation. One-step SLK procedures for polycystic disease were introduced in 08/2013 at IKEM Prague (n = 6) and 11/2014 at University Hospitals Leuven (n = 6). Feasibility and safety of the one-step technique were investigated. We compared surgical data and outcomes obtained with the one-step technique to all consecutive two-step procedures performed for polycystic disease at the University Hospitals Leuven between 2008–2014 (n = 23). Median (interquartile range) are given. One-step SLK offered broad and adequate exposure for the hepatectomy, nephrectomies and transplantations, which were all uneventful. Morbidity, patient (100% vs 91%, p = 0.53) and graft survival (100% graft survival for liver and kidney in both groups) were comparable between one-step and two-step SLK. Liver cold ischaemia time was comparable [6.0 (4.4–7.6) vs. 7.1 (3.9–7.3), p = 0.077], kidney cold ischaemia time was shorter in one-step compared to two-step SLK [8.1 (6.4–9.3) vs. 11.7 (10.0–14.0), p<0.001)]. Total procedural time was also shorter in one-step compared to two-step SLK [6.8 (4.1–9.3) vs. 9.0 (8.7–10.1), p = 0.032], while all underwent bilateral (67%) or unilateral (33%) nephrectomy (compared to 0% and 52% in two-step SLK, respectively). In one-step SLK, 67% received a pre-emptive kidney transplant compared to 46% in two-step SLK. 5/12 two-step SLK became dialysis dependant after pre-transplant nephrectomy, the 4 dialysis-dependant patients with one-step SLK had not undergone pre-transplant nephrectomy. In conclusion, one-step SLK for polycystic disease is feasible and safe.

## Introduction

Since the first report on simultaneous liver kidney transplantation (SLK) by Margreiter et al [1], SLK has become an accepted treatment for end-stage liver and renal failure in adults as it has been shown to provide good graft and patient outcome [2]. Polycystic liver and kidney disease is a frequent and increasing indication for SLK [3]. In Eurotransplant, 32% of SLK were performed for polycystic disease in 2004, increasing to 51% in 2013 [4].

Like the first reported SLK (R. Margreiter, personal communication), the transplant is classically performed as a two-step procedure with the kidney following the liver transplantation. The liver is transplanted through any form of right subcostal incision, often a Mercedes incision that opens the abdomen via a bilateral subcostal incision with a midline extension up to the xiphoid process [5]. After skin closure the patient is usually re-draped and the kidney is transplanted through a separate left or right oblique Rutherford Morison or Alexandre incision in the preperitoneal space [6]. In polycystic disease, this classical two-step technique can be very challenging as the liver hilum has a more caudal location due to the liver volume and the standard subcostal incision does not always allow ideal exposure. Furthermore, there is a frequent need for uni- or bilateral nephrectomy in these patients, either to create space for the kidney graft, to remove any focus of infection in renal cysts, or because the patient suffers pain caused by organ volume. A nephrectomy, especially of voluminous organs, is challenging when performed through a (bilateral) subcostal incision -even with lateral extensions of the incision- but facilitated by a midline laparotomy.

An alternative technique for SLK is a one-step procedure where the transplantations are performed through a xiphopubic laparotomy and a preperitoneal pocket for the kidney graft is created. Since the introduction of the one-step SLK technique in the Institute for Clinical and Experimental Medicine (IKEM) in Prague (08/2013) and the University Hospitals Leuven (UZL) in Belgium (11/2013), all SLK for polycystic disease have been performed by the one-step approach. Although the use of a midline laparotomy in SLK has anecdotally been mentioned in the literature[7–9], the technique has never been described in detail nor has it been compared to the classical two-step approach.

We therefore describe the technique of a one-step SLK through a midline laparotomy and report on the feasibility, safety, and outcome of the procedure in comparison with the two-step approach.

## Methods

### Clinical data and statistical methods

Donor, recipient, surgical, and follow-up data of 12 patients undergoing SLK by midline laparotomy for polycystic disease in IKEM and UZL were retrieved from prospectively kept databases. The occurrence of surgical postoperative complications (Clavien-Dindo III or higher) within 90 days post-transplant was retrieved from patient records. Donor, recipient, transplant characteristics and outcomes were compared to those of all (n = 24) consecutive SLK performed by the two-step procedure for the same indication at UZL between 2008–2014. In one case of the 24 consecutive two-step procedures, the kidney transplantation did not immediately follow the liver transplantation and this case was excluded from the comparison. Procedural time was defined as the time from (first) skin incision until closure of the (last) wound. Continuous data are presented as median (interquartile range), categorical data as number (percentage). Mann-Whitney U tests, Fisher exact or Chi square tests were used, p<0.05 was considered significant (GraphPad Prism 5 for Windows). Patients gave written informed consent to the use of their data for research purposes. Patients also consented to the use of photographical images. The study was approved by the medical ethical committee of UZL (s59327).

### Technique of the two-step SLK

In the historical comparator group all transplantations were performed through a bilateral subcostal incision after preparation of the organs on the back-table. The liver was implanted using caval replacement and veno-venous bypass was routinely used in all cases. If a nephrectomy was performed during transplantation, this was done during the anhepatic phase with the patient on full bypass. Kidney transplantation followed the liver transplantation as a separate procedure and was performed with the kidney in either left or right iliac fossa and placed in a preperitoneal pocket as classically described [6]. The implantation site for the kidney was determined preoperatively and cannulation for the veno-venous bypass was done on the contra-lateral side of the planned kidney implantation site.

### Technique of the one-step SLK through a xiphopubic incision

**Fig 1:**
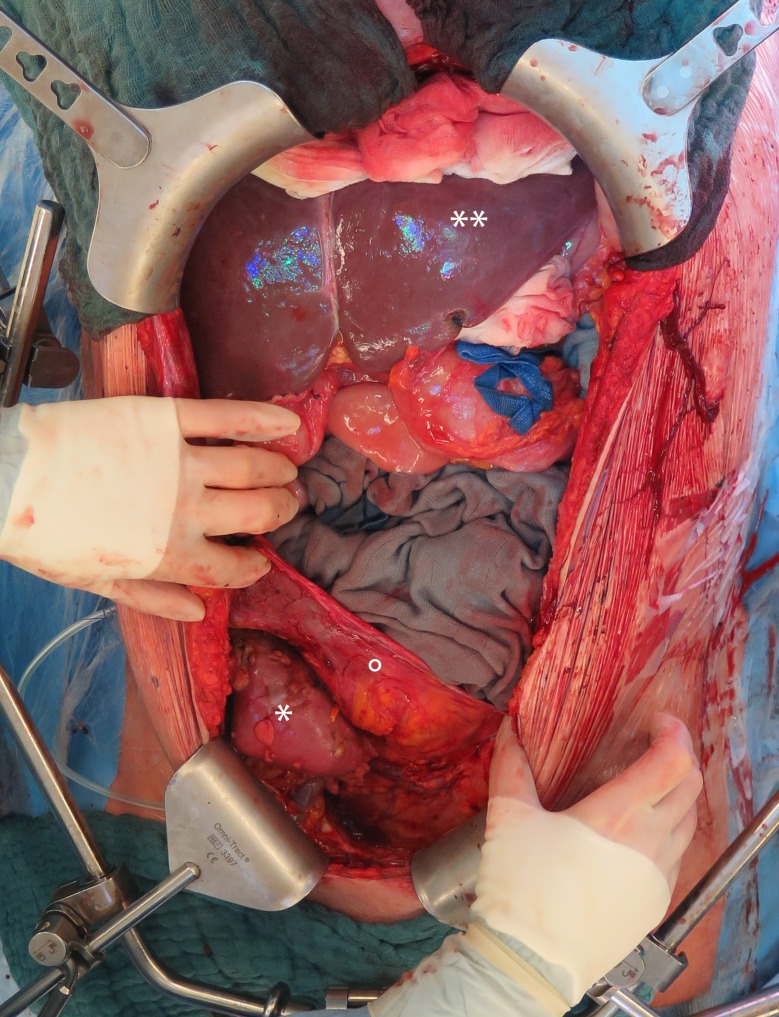
Intraoperative view of the liver and kidney graft after simultaneous liver and kidney transplantation with native nephrectomy. Midline incision with cephalad retraction of the ribs showing the liver graft (**) and the kidney graft (*) in the right iliac fossa where a preperitoneal pocket (°) was created from the midline. This pocket is closed by taking the peritoneum with the sutures closing the midline.

Liver and kidney grafts were prepared for implantation on the back-table as usual. The abdomen was opened through a xiphopubic incision. Exposure of the liver was facilitated by bilateral cephalad retraction of the ribcage. Lateral retraction of the abdominal wall exposed the rest of the abdomen (Fig 1). In this way, liver, native kidneys, and both iliac fossae were easily accessible.

Liver transplantation then followed its usual course with either a classical caval replacement technique (UZL, as described above) with the routine use of veno-venous bypass or a piggy-back technique (IKEM) without the use of a temporary portocaval shunt. If a nephrectomy was performed, this was done either during the anhepatic phase or after reperfusion of the liver (S1 Table).

**Fig 2:**
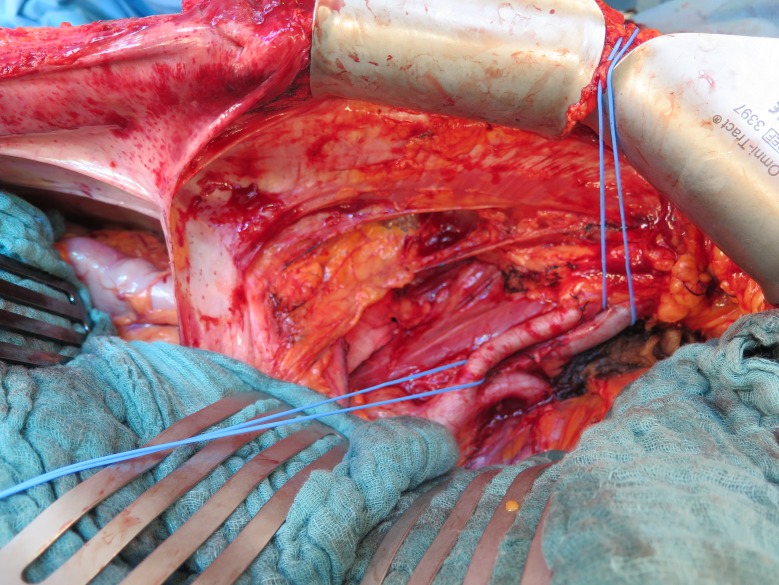
Intraoperative view on the iliac fossa after creation of the preperitoneal flap. Exposure of the iliac vessels through a midline incision after the creation and cephalad retraction of the peritoneal flap (°), in this case created by peeling it down from the midline.

**Fig 3:**
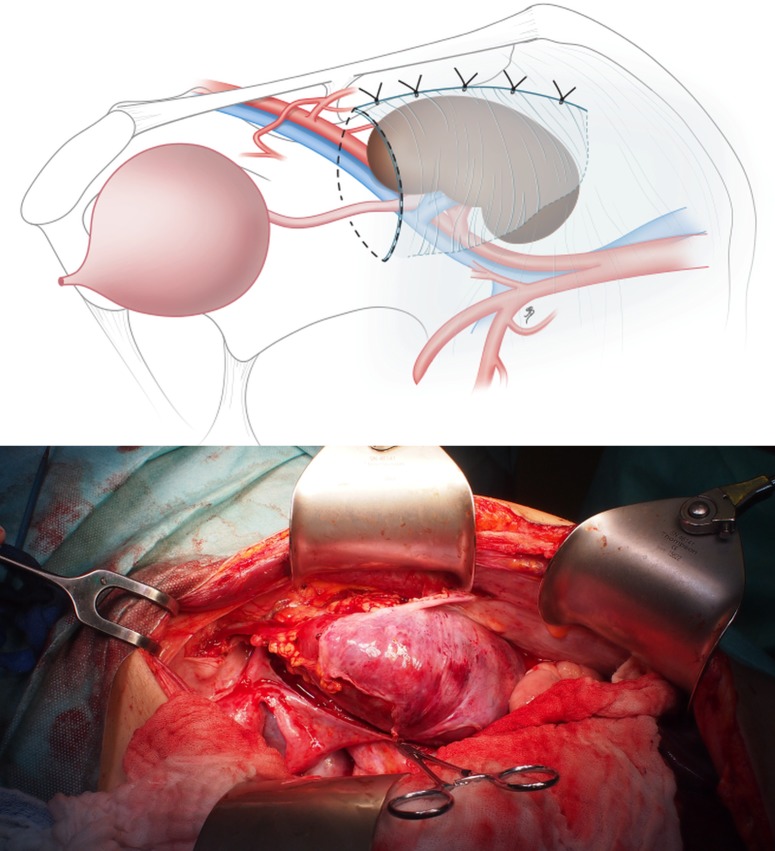
Illustration and intraoperative view showing the approach to an L-shaped incision to create a preperitoneal flap. An alternative to create the preperitoneal pocket for the kidney graft starting from a separate L-shaped incision of the peritoneum that is flipped down later and closed separately after the kidney transplantation.

The kidney graft is placed in a preperitoneal pocket (15x15cm). This pocket can be created by peeling down the peritoneum from the midline (Fig 1) or from a separately made L-shaped peritoneal incision (Fig 2). After the creation of this pocket, the iliac vessels (Fig 3) and bladder are prepared and the kidney is transplanted using the classical technique. The peeled down peritoneum is closed either by taking it into the sutures that close the laparotomy or by closing the L-shaped incision separately.

## Results

The one-step approach for SLK in polycystic disease was introduced at UZL after observing the technique at IKEM where it has been in use since 08/2013. Twelve cases with a median follow-up of 373 days (120–684) have been performed between those dates and 06/2016 (6 IKEM, 6 UZL). In each case, broad and adequate exposure was obtained for the hepatectomy, nephrectomies, and transplantations which were all uneventful. Eight one-step SLK were accompanied by a bilateral nephrectomy, in the other four cases a unilateral nephrectomy was performed (which in two patients followed a previous nephrectomy) (Table 1). The creation of the preperitoneal pocket during one-step SLK did not cause any difficulties and was not compromised by native kidney nephrectomy.

**Table 1:** Timing of nephrectomy.

Demographics	One-step SLK	Two-step SLK
**Number recipients; n**	12	23
**No nephrectomy performed; n (%)**	0 (0)	5 (22)
**Pretransplant nephrectomy; n (%)**	2 (22)	8 (35)
**bilateral**	0 (0)	4 (17)
**unilateral**	2 (22)	4 (17)
right	1	2
left	1	2
**Simultaneous nephrectomy; n (%)**	12 (100)	12 (52)
**bilateral**	8 (67)	0 (0)
**unilateral**	4 (33)	12 (52)
right	2	11
left	2	1
**Posttransplant nephrectomy; n (%)**	0 (0)	1 (4)

Table 2 shows the demographical data of donors, recipients, surgery, and outcome of one-step and two-step SLK procedures. S1 Table shows all information per patient. There were no differences in donor and recipient age, gender distribution, and body mass index (BMI). The Model of End Stage Liver Disease (MELD) score was slightly, but not statistically, lower in patient undergoing a midline laparotomy though median MELD was similar. Preoperative need for blood product transfusion was similar in one-step and two-step SLK (Table 2).

**Table 2:** Donor, recipient, and transplantation demographics and outcome data.

Demographics	One-step SLK	Two-step SLK	p-value
Number	12	23	
Follow-up (d)	373 (120–684)	1455 (1029–2069)	0.76
Donor age (y)	39 (28–58)	51 (37–56)	0.53
Donor gender (M/F); n (%)	9/3 (75/25)	12/11 (52/48)	0.28
Recipient age (y)	55 (45–61)	60 (53–62)	0.16
Recipient gender (M/F); n (%)	1/11 (8/92)	4/19 (17/83)	0.64
Recipient BMI (kg/m^2^)	25 (23–26)	23 (21–26)	0.19
Lab MELD	20 (13–20)	20 (20–21)	0.023
Pre-emptive kidney transplant; n (%)	8 (67)	11 (46)	0.30
Cold ischaemia time liver (h)	6.0 (4.4–7.6)	7.1 (3.9–7.3)	0.077
Cold ischaemia time kidney (h)	8.1 (6.4–9.3)	11.7 (10.0–14.0)	<0.001
Time incision to closure (h)	6.8 (4.1–9.3)	9.0 (8.7–10.1)	0.032
Perop transfusion			
Packed red blood cells (U)	3 (1–5)	3 (1–5)	0.76
Fresh frozen plasma (U)	3 (0–11)	4 (0–6)	0.81
Platelets (pools)	0 (0–0)	0 (0–0)	0.17
Length of ICU stay (d)	5 (2–7)	3 (2–4)	0.10
Length of stay (d)	18 (13–30)	16 (13–24)	0.34
Occurrence incision hernia; n (%)	0 (0)	4 (17%)	0.28
Graft survival liver*; n (%)	12 (100)	21 (100)	-
Graft survival kidney*; n (%)	12 (100)	21 (100)	-
Patient survival; n (%)	12 (100)	21 (91)**	0.54

Continuous data are presented as median (interquartile range), categorical data as number and percentage.

*censored for patient death

** died with functioning grafts. BMI, body mass index; F, female; ICU, intensive care unit; M, male; MELD, model of end stage liver disease; U, units.

Cold ischaemia time of the liver was comparable between one-step and two-step SLK (Table 2). Cold ischaemia time of the kidney and total procedural time were at median 3.6h and 2.2h shorter when a one-step SLK was performed while 67% of them underwent a bilateral nephrectomy and 33% a unilateral nephrectomy (compared to 0% and 48% in the two-step SLK, respectively) (Table 2).

In the cohort of one-step SLK, 67% of patients were pre-emptively transplanted compared to 46% in the two-step SLK cohort (p = 0.30). Of the 11 patients on dialysis at time of two-step SLK, 5 became dialysis dependent after a previous nephrectomy, while the 4 patients on dialysis at time of one-step SLK had not undergone a nephrectomy before transplantation (S1 Table).

All patients recovered well and all grafts functioned immediately. The occurrence of surgical complications Clavien-Dindo grade III or higher within 90 days after transplantation was similar in the one-step and two-step SLK (Table 3). Three anastomotic biliary strictures occurred (2 in one-step, 1 in two-step) and were managed by endoscopic retrograde cholangiography or percutaneous transhepatic cholangiography and stenting. One ureteral leak was managed with ureteral stenting after one-step SLK. A pancreatic fistula occurred after one-step SLK in a patient that had a history of a total gastrectomy where a small pancreas laceration had occurred during hepatectomy. The fistula was surgically drained. Length of stay in the intensive care unit and the hospital was similar in one-step and two-step SLK. No incisional hernias have developed during follow-up in one-step SLK, compared to 4/23 in the two-step SLK at a median follow-up of 382d (172–678). Patient and graft outcome was similar between one-step and two-step procedure (Table 1) with 2 patient deaths in the two-step SLK (one early death due to invasive aspergillosis, one 2.5y post-transplant as a consequence of a newly developed non-small cell lung carcinoma.

**Table 3:** Complications Clavien-Dindo grade ≥ III during the first 90 days after simultaneous liver kidney transplant.

Complication	One-step SLK	Two-step SLK	p-value
Any complication **≥ III**	3	3	0.37
Grade III	3	2	0.19
Grade IV	0	0	-
Grade V	0	1	0.46

## Discussion

This limited series shows that a one-step SLK through a midline laparotomy for SLK in polycystic disease is feasible and safe compared to the classically used two-step procedure.

Morbidity and mortality were comparable between one-step and two-step procedures but shorter kidney cold ischaemia times and procedural times were reached in the one-step SLK, even though in all cases a uni- or bilateral nephrectomy was performed during the one-step SLK. The reduced operative times are likely due to a combination of factors among which the fact that only one incision is made and only one needs to be closed. Other logistical factors, e.g. opening of new draping and instrument sets, re-draping of the patient, … might also influence procedural times.

In all cases a (bilateral) nephrectomy was performed to either create space for the kidney graft, to remove any potential focus of infection, or to alleviate symptoms of pain or haemorrhagic cyst complications. The midline approach in the one-step SLK facilitated exposure and nephrectomies were performed without complications. The nephrectomy did not compromise the creation of the preperitoneal pocket.

A nephrectomy can also be performed during the two-step procedure but this is often difficult. A right nephrectomy can be performed during liver transplantation through a subcostal incision, and this is frequently done (as it was in 11/23 of the two-step SLK patients). Nevertheless, it can be challenging, especially if the organ is voluminous. Performing a left nephrectomy during liver transplantation through a subcostal incision is even more challenging, even with a left lateral extension of the incision, and this was performed only once in the two-step SLK patients. Uni- or bilateral nephrectomy can be performed independent of the transplantation through a midline laparotomy (i.e. before or after SLK depending on the indication and patient characteristics, as was done in 9/23 two-step SLK cases), exposing the patient to increased operative risks. If nephrectomy is performed pre-transplant there is a considerable chance the patient becomes dialysis dependent if not already on renal replacement therapy. Furthermore, and as recently described for live donor kidney transplants with simultaneous bilateral nephrectomy for polycystic disease, a combined operative approach avoids multiple procedures, dialysis, and costs of staged operations [10].

Although graft and patient survival are the most important outcomes, postoperative morbidity like pain, wound infections, incisional hernia and evisceration complicate recovery and increase costs. Any surgical incision should provide access and exposure while minimizing morbidity. In elective surgery, transverse incisions cause less pain, wound infections, incisional hernias and pulmonary dysfunction [11]. As the transplant population is more prone to any of these, transverse incisions are attractive. However, there is no literature on pain and functional recovery after two transverse incisions (in this case a subcostal for the liver and an oblique incision in the iliac fossa for the kidney), especially in the fragile immunosuppressed transplant population. Furthermore, when these incisions are both on the right, transecting superior and inferior epigastric bundles compromises muscle and skin vascularization. Placing the kidney on the left would avoid this but multiple factors determine implantation site and do not always favour the left fossa.

Midline incisional hernias, although perhaps more frequent, are easier to repair with more options available in case of recurrence. Also, diligently following the newest guidelines on abdominal wall closure (single layer, continuous slowly absorbable sutures, suture to wound length ratio of four or more, and short stitch length) will likely reduce the incidence of incisional hernias [12, 13]. We did not see an increased incidence of incisional hernias or evisceration after a midline laparotomy compared to patients who had an SLK by a two-step procedure, although follow-up in the one-step SLK was shorter and incisional hernias might still develop.

The preperitoneal position of the kidney graft allows ultrasound-guided biopsies with decreased risk of intra-abdominal bleeding or injury to a bowel. This is why we place the kidney in a preperitoneal pocket during one-step SLK. We have found the creation of a preperitoneal pocket to be an elegant technique in other intra-abdominal simultaneous transplants such as pancreas-kidney and small bowel-kidney as well.

The two-step approach remains particularly interesting when hemodynamic instability, coagulation disorders etc. are anticipated in which case the kidney transplantation can be postponed for a few hours until the patient is more stable. Another indication would be a positive cross-match or a highly sensitized recipient where postponing the kidney transplantation allows time for the liver to absorb circulating antibodies [14]. These conditions aren’t necessarily known at the time of incision but a midline laparotomy does not commit the surgeon to an SLK by the one-step approach. The surgery can be started by limiting the midline laparotomy to the umbilical level and extending it to the pubis once the final decision to pursue the kidney transplant has been made. Should the team feel the kidney transplant is best postponed for a few hours, a sequential classical hockey stick incision could be made.

One might wonder whether the midline SLK approach could be used for indications other than polycystic disease as indeed we have performed one case of SLK for alcoholic cirrhosis with dialysis dependent chronic renal failure in a non-obese patient with good exposure. Although there are no data in the literature, the technique might not provide much benefit in obese patients or those without ascites.

As the comparator group consisted only out of UZL patients, there are differences in perioperative care and logistics that might have influenced the results. Although both groups had comparable baseline characteristics, these limitations need to be kept in mind when interpreting these data and these initial results need to be confirmed in larger series and ideally in a randomised controlled trial.

## Conclusion

A midline approach for SLK in polycystic disease allows feasible and safe access with excellent exposure and can be combined with uni- or bilateral nephrectomy, potentially avoiding pretransplant renal replacement therapy. Furthermore, the technique shortens procedural and kidney cold ischaemia times. Preperitoneal placement of the kidney is easily performed by peeling down the peritoneum from the midline or a separate L-shaped peritoneal incision. However, more prospective data on morbidity of various surgical approaches for SLK in polycystic disease and other indications are needed to verify these initial findings.

## Supporting information

S1 Table Source data. (XLSX)
